# The Relationship of the FOUR Score to Patient Outcome: A Systematic Review

**DOI:** 10.1089/neu.2018.6243

**Published:** 2019-08-20

**Authors:** Ching C. Foo, James J.M. Loan, Paul M. Brennan

**Affiliations:** ^1^ College of Medicine and Veterinary Medicine, University of Edinburgh, Edinburgh, United Kingdom.; ^2^ Centre for Clinical Brain Sciences, University of Edinburgh, Edinburgh, United Kingdom.

**Keywords:** coma, consciousness, FOUR score, full outline of unresponsiveness, outcome, systematic review

## Abstract

The Full Outline of UnResponsiveness (FOUR) score assessment of consciousness replaces the Glasgow Coma Scale (GCS) verbal component with assessment of brainstem reflexes. A comprehensive overview studying the relationship between a patient's FOUR score and outcome is lacking. We aim to systematically review published literature reporting the relationship of FOUR score to outcome in adult patients with impaired consciousness. We systematically searched for records of relevant studies: CENTRAL, MEDLINE, EMBASE, Scopus, Web of Science, ClinicalTrials.gov, and OpenGrey. Prospective, observational studies of patients with impaired consciousness were included where consciousness was assessed using FOUR score, and where the outcome in mortality or validated functional outcome scores was reported. Consensus-based screening and quality appraisal were performed. Outcome prognostication was synthesized narratively. Forty records (37 studies) were identified, with overall low (*n* = 2), moderate (*n* = 25), or high (*n* = 13) risk of bias. There was significant heterogeneity in patient characteristics. FOUR score showed good to excellent prognostication of in-hospital mortality in most studies (area under curve [AUC], >0.80). It was good at predicting poor functional outcome (AUC, 0.80–0.90). There was some evidence that motor and eye components (also GCS components) had better prognostic ability than brainstem components. Overall, FOUR score relates closely to in-hospital mortality and poor functional outcome. More studies with standardized design are needed to better characterize it in different patient groups, confirm the differences between its four components, and compare it with the performance of GCS and its recently described derivative, the GCS-Pupils, which includes pupil response as a fourth component.

## Introduction

Clinicians' management decisions about acute traumatic brain injury (TBI) patients are guided by assessments of the person's current state and may also be influenced by their perceptions of its relation to the patient's likely outcome.^1^ Internationally, the Glasgow Coma Scale (GCS) is the most widely used tool for assessing and communicating about a patient's responsiveness.^2^ All the three components—eye, motor, and verbal responses—relate to outcome,^3^ as does the derived summation into the GCS score, albeit with some loss of information. Moreover, the GCS is combined with other features, such as pupil response, age, and injury characteristics, in numerous multi-variate prognostic models for predicting functional outcome and mortality.^4–6^ The difficulty in assigning a verbal response in an intubated patient and the separation of assessment of brain stem features, such as pupil response, in multi-variate modeling stimulated specialists in neurological intensive care to propose an alternative approach.

The Full Outline of UnResponsiveness (FOUR) score was described by Wijdicks and colleagues. It is based on the eye and motor components of the Glasgow system, but the verbal component was removed and two new components added, namely brainstem reflexes and respiratory pattern. The FOUR score was developed for the assessment of level of consciousness in patients admitted to a neurointensive care unit.^7^ This was with the purpose of improving the standardized assessment of level of consciousness for patients who are intubated or have focal neurological deficits. Each component is a 5-point scale, ranging from 0 to 4, with combined FOUR score ranging from 0 to 16, with 16 indicating the highest level of consciousness. Unlike the GCS, the eyes must be able to track or blink to command in order to obtain the maximum score of 4 points for eye component in FOUR score. Table 1 shows the scoring criteria for all components of FOUR score and GCS. The FOUR score approach emphasizes description of a patient by the combined score and the validity of the latter as an index of acute severity through its relationship to outcome. In order to provide a comprehensive assessment of the latter, we have performed a systematic review of the reported evidence, with focus on prognostic performance in groups of patients particularly targeted by FOUR score, namely those with a neurological diagnosis, intubated patients, and those admitted to dedicated neuroscience centers.

**Table 1.** Components of the FOUR Score and Glasgow Coma Scale

*Full Outline of UnResponsiveness Score*	*Glasgow Coma Scale*
Eye response 4 = eyelids open or opened, tracking, or blinking to command 3 = eyelids open but not tracking 2 = eyelids closed, but open to loud voice 1 = eyelids closed, but open to pain 0 = eyelids remain closed with pain	Eye opening 4 = spontaneous 3 = to speech 2 = to pain 1 = none
Motor response 4 = thumbs-up, fist, or peace sign 3 = localizing to pain 2 = flexion response to pain 1 = extension response to pain 0 = no response to pain or generalized myoclonus status	Best motor response 6 = obeying commands 5 = localizing to pain 4 = withdrawal from pain 3 = abnormal flexion response to pain 2 = extension response to pain 1 = none
Brainstem reflexes 4 = pupil and corneal reflexes present 3 = one pupil wide and fixed 2 = pupil or corneal reflexes absent 1 = pupil and corneal reflexes absent 0 = absent pupil, corneal and cough reflex	Verbal response 5 = orientated 4 = confused 3 = inappropriate words 2 = incomprehensible sounds 1 = none
Respiration 4 = not intubated, regular breathing pattern 3 = not intubated, Cheyne-Stokes breathing pattern 2 = not intubated, irregular breathing 1 = breathes above ventilator rate 0 = breathes at ventilator rate or apnea	

FOUR, Full Outline of UnResponsiveness.

## Methods

The protocol for this review was registered at PROSPERO (ID: CRD42017065443). The methodology and report of this review were prepared based on the Preferred Reporting Items for Systematic Reviews and Meta-Analyses (PRISMA) statement.^8^

### Eligibility criteria

All prospective, observational studies and randomized, controlled trials published between 2005 and 2018 were considered for inclusion. Studies were included if they reported data on patients with impaired consciousness of any cause, where level of consciousness was assessed using FOUR score, and where the outcome was reported in terms of mortality or a validated measure of functional outcome, such as modified Rankin Scale (mRS)^9^ or Glasgow Outcome Scale (GOS).^10^ To permit analysis of predictive power of FOUR score at various time points, studies reporting any time points of outcome were eligible for inclusion. Studies were excluded if they were abstracts, commentaries, letters, correspondences, reviews, and if the full-text article was not available or was not written in the English language. Studies involving pediatric patients, where the mean or median age of sample population was <18 years of age, were also excluded.

### Information sources and search strategy

The following electronic databases were searched: The Cochrane Central Register of Controlled Trials (CENTRAL), MEDLINE (PubMed), EMBASE, Scopus (www.scopus.com), Web of Science (www.webofknowledge.com), and ClinicalTrials.gov (www.clinicaltrials.gov).

Gray literature searching was performed using the OpenGrey database (www.opengrey.eu). The search was limited to articles listed between 2005 and April 2018 (inclusive) and was last conducted on July 22, 2018. To maximize sensitivity of our search strategy, the title and abstract of references were searched for the keywords “full outline of unresponsiveness” OR “four score.” No AND operators were used. Citation searching was not performed. The search strategy used for each database is in (Supplementary Table S1).

### Data management, selection, and extraction

Citations were de-duplicated using Mendeley reference management software (version 1.17.7; Mendeley Ltd., London, UK) before importing and screening using Covidence (www.covidence.org). Two authors independently reviewed titles and abstracts against the eligibility criteria. For abstracts which were potentially eligible, or if eligibility was unclear from abstract review, full texts were examined independently by both authors and any disagreement resolved by consensus. Reasons for exclusion at the phase of full-text review were recorded. Data extraction from included studies was performed independently by two authors using a standardized proforma as per the protocol, with any discrepancies resolved by the third author. Data items for extraction are described in the supplementary materials (Supplementary Table S2).

### Assessment of risk of bias of study

Risk of bias of each study was assessed independently by two authors using the Quality In Prognosis Studies (QUIPS) tool,^11^ which assesses risk of bias in six domains—study participation, study attrition, prognostic factor measurement, outcome measurement, study confounding, and statistical analysis and reporting. Any disagreement was resolved by discussion. For the purpose of summarization of data, studies were globally rated “low risk of bias” if all the components were rated “low risk”; “moderate risk of bias” if one or more components were rated “moderate risk”; and “high risk of bias” if one or more components were rated “high risk.”

### Data synthesis

The characteristics of included studies and risk of bias assessment results are described in tables. The studies were categorized according to the outcomes measured (mortality, GOS or extended Glasgow Outcome Scale–Extended [GOSE],^12^ mRS, and others). Timing of assessment in each study was recorded. Measures of predictive ability of FOUR score for each outcome are presented in terms of area under receiver operating characteristic curve (AUC), classified into several performance levels,^13^ or odds ratio of mortality or poor outcome associated with each increment in total FOUR score, as determined by logistic regression. On the basis of analyzing receiver operating characteristics curve, optimal predictive values of FOUR score in terms of maximum sensitivity and specificity were identified. This is termed the “cut off.” We describe sensitivity and specificity reported by each included study at their reported cut-off value. Results based on total FOUR score are presented, unless stated otherwise.

In addition, further comparisons regarding the prognostic ability of FOUR score were made in three subgroup analyses between: 1) patients with neurological and non-neurological causes of impaired consciousness; 2) patients in specialized neurological units and non-neurological units; and 3) intubated and non-intubated patients.

These were broadly categorized because the review is limited to data presented by published studies which differed methodologically, thus unlikely to yield sufficient studies of similar methodology for more-specific subgroup analyses. Subgroup analysis of severity of impairment of consciousness was considered but not performed given that studies have included population of full range of consciousness in statistical analysis, thus access to raw data would be necessary to perform this analysis.

I^2^ value was calculated using MedCalc software (version 17.5.5; MedCalc Software bvba, Ostend, Belgium), with I^2^ value >50% considered as significant heterogeneity. The high level of statistical heterogeneity (I^2^ = 84.9%) between studies, calculated using AUC values, and methodological heterogeneity, particularly of timing of initial assessment and outcome assessment,^14–23^ precluded meta-analysis. We therefore conducted a narrative synthesis alone.

## Results

**FIG. 1.**
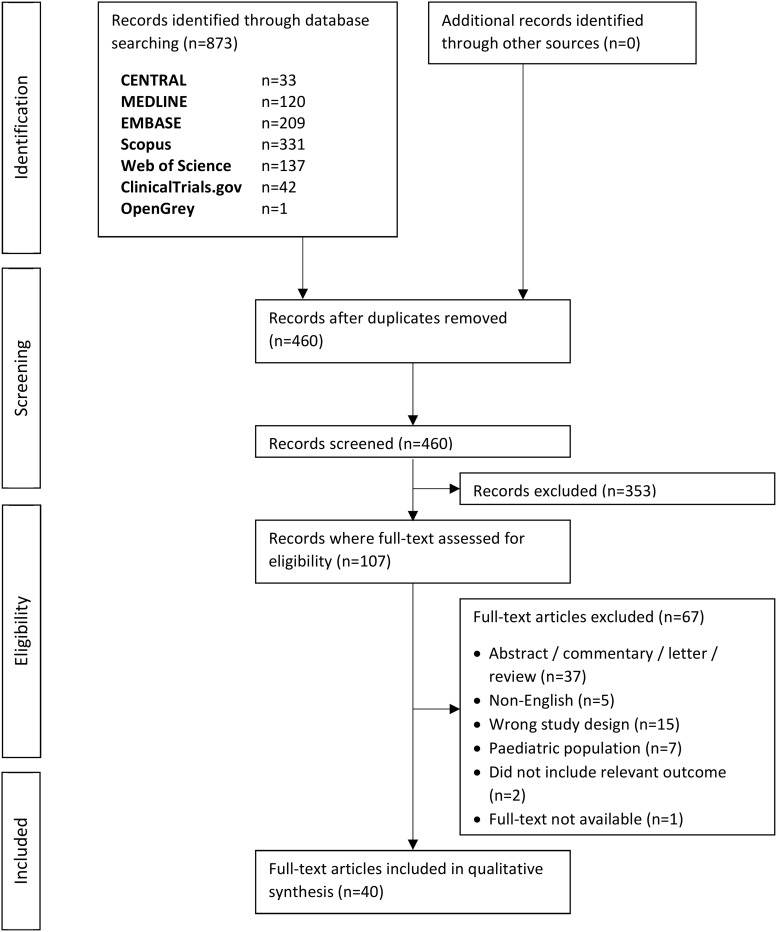
Flow diagram of the study selection process.

In total, 460 records were identified from the literature search after de-duplication. After title and abstract screening, 107 records were selected for full-text review, of which 67 records were excluded (36 abstracts, one commentary, 15 did not meet study design criteria, seven studies on pediatric population, two studies without acceptable outcome, five non-English articles, and one record where the full-text article was not available despite a rigorous search online). Finally, 40 records were included. The summary of the study selection process is illustrated in the flow diagram (Fig. 1).

### Characteristics of studies

Among the 40 records included in this review (39 cohort studies, one case-control^24^), most studies were conducted in the intensive care unit (ICU; *n* = 27), and among these, six were specialized neurological or neurosurgical ICUs (Table 2). In total, reports of 5767 patients were included. The number of patients reported by each included study ranged from 35^16^ to 1645.^15^ For the purposes of this review, reports by Gorji and colleagues^16,25^ and Hosseini and colleagues^26^ of a single population of 80 patients were considered to be a single study. Likewise, reports of a single cohort by McNett and colleagues in 2016^27^ and 2014^17^ were considered to be a single study. The reported mean age of participants in included studies varied between 32^18^ and 70^28,29^ years of age.

**Table 2.** Characteristics of Included Studies (*n* = 40)

				*Causes^b^*								
	*Setting^a^*	*No. of patients*	*Mean age, years*	*Neuro*	*Non-neuro*	*% of patient intubated^c^*	*Observer^d^*	*Assessment timing from admission^e^*	Mean/median^*^ FOUR score	*Outcome^f^*	*Outcome timing^g^*	*Overall RoB*
Akavipat 2011^20^	Ns	304	53.2	100%	0%	—	P, N	0–30min	—	Mortality, GOS	Dc	Mod
Babu 2017^40^	ED, N.ICU	98	34.7	100%	0%	94%	R	0–1d	11.26	Mortality	Dc	High
Baratloo 2016^18^	ED	89	32	96.6%	3.4%	—	R	adm, 6h, 12h	10.9	Mortality	Dc	High
Bruno 2011^43^	MG.ICU	176	63	94.9%	5.1%	74%	N, NPsy, ICU Sp, R	0–1mo	—	GOS	3mo	Mod
Chen 2013^44^	N.ICU	101	64	100%	0%	91%	Ne	0–1d	5.6	Mortality, GOS	30d	Mod
Eken 2009^14^	ED	185	med^*^ 59	80%	20%	0%	P, R	adm	—	Mortality, mRS	3mo	Low
Fischer 2010^31^	ICU	267	63	32.2%	67.8%	22.5%	Ne, N, P	0–3d	—	Mortality	28d	Mod
Fugate 2010^33^	N/S	136	62	0%	100%	—	NIn	1–2d, 3–5d	—	Mortality, CPC	Dc	High
Gorji 2014^16^	ICU	35^†^	34	100%	0%	—	Iv	0–24hr	—	Mortality, GOS	Dc	High
Gorji 2015^25^	ICU	80^†^	34	100%	0%	100%	Iv	0–24hr	—	Mortality	<14d, >14d	High
Gujjar 2013^45^	M.W, M.HDU	100	62	58%^¶^	60%^¶^	0%	Ne, R, SHO	0–24hr	13	Mortality, mRS	Dc, 3mo	Mod
Hosseini 2017^26^	ICU	80^†^	34	100%	0%	—	—	0–24hr	—	Mortality	<14d, >14d	High
Hu 2017^48^	N.ICU	102	65	100%	0%	63%	—	3d	10 (good recovery) 6 (poor recovery)	Awareness rec.	90d	Mod
Iyer 2009^46^	ICU	100	63	55%	45%	—	N, F, Con^§^	N/S	—	Mortality, mRS	Dc, 3mo	Mod
Jalali 2014^38^	ICU	104	41	100%	0%	0%	N	0–1d	—	Mortality	Dc/≤14d	High
Kasprowicz 2016^35^	ICU	162	med^*^ 52	100%	0%	83%	—	0–24hr, ICU Dc	6^*^	Mortality, GOS	Dc, 3mo	Mod
Khanal 2016^21^	ICU	97	—	100%	0%	—	—	0–24hr	7.89	Mortality	Dc	High
Kocak 2012^28^	N.ICU	100	70	100%	0%	—	Ne	adm, 1d, 3d, 10d	11.9 (survivors) 9.6 (non-survivor)	Mortality	Dc	High
Lee 2017^49^	ED	105	68.3	100%	0%	—	P, N	0–1hr	16^*^	Mortality	Dc	Mod
Mansour 2015^22^	CCU	127	62	100%	0%	—	—	24h, 72h	—	Mortality, mRS	Dc/30d, 3mo	Mod
Marcati 2012^29^	N, Ns, ICU, ED	87	70	100%	0%	17%	Ne, R	0–7d	—	Mortality, mRS	Dc	Mod
McNett 2014^17^	S.ICU	136^‡^	53	100%	0%	—	Iv	24hr, 72hr	15^*^	Mortality	Dc	High
McNett 2016^27^	ABIC	107^‡^	54	100%	0%	—	Iv	24hr, 72hr	15^*^	GOS	6mo, 12mo	Mod
Momenyan 2017^41^	ICU	84	42.6	100%	0%	72%	P, N, Stu	0–7d	—	Mortality, mRS	Dc	Mod
Okasha 2014^19^	ED	60	med^*^ 29	100%	0%	78%	In	adm	11^*^	GOSE, ED intub	Dc/28d, 1mo	Mod
Peng 2015^23^	Ns.ICU	120	48	100%	0%	48.30%	NsR, N	0–1d	—	Mortality, mRS	Dc, 3mo	Mod
Rohaut 2017^34^	ICU	148	67.4	0%	100%	100%	P	24hr post-sed	4	Mortality	28d	Low
Sadaka 2012^36^	N.ICU	51	58	100%	0%	—	Iv	0–24hr	13^*^	Mortality, mRS, GOS	Dc, 3–6mo	Mod
Said 2016^30^	ICU	86	med^*^ 63	15.10%	84.90%	100%	P	≤24hr intub	8.5^*^	Mortality, extub, GOS	28d, 14d, 3mo	Mod
Saika 2015^37^	ED	138	38	100%	0%	—	Iv	adm	11	Mortality	14d	High
Senapathi 2017^42^	ED, ICU	63	med^*^ 32	100%	0%	—	—	adm, 24hr, 48hr, 72hr	—	Mortality	Dc	High
Sepahvand 2016^39^	ICU	198	41	100%	0%	—	N	24–48hr	—	Mortality	Dc	Mod
Stead 2009^47^	ED	69	—	100%	0%	—	P, R, N	N/S	16^*^	Mortality, mRS	Dc	Mod
Surabenjawong 2017^50^	ED	60	med^*^ 65	100%	0%	8.3%	P, R	adm	14.05	Mortality, mRS, CPC	3mo	Mod
Weiss 2015^32^	ICU	85	60	5.9%	94.1%	100%	In	Δ3d–1d	—	Mortality, CPC	6mo	Mod
Wijdicks 2005^7^	ICU	120	59	100%	0%	47.50%	In, R, N	0–1d	—	Mortality, mRS	Dc, 3mo	Mod
Wijdicks 2015^15^	ICU	1645	60	29.5%^¶^	76.6%^¶^	32.80%	P	0–1hr	—	Mortality	Dc	Mod
Wolf 2007^24^	ICU	80	64	100%	0%	N/S	N	0–24hr	—	Mortality, mRS	Dc, 30d	Mod
Zappa 2017^51^	ICU	40	64.4	100%	0%	100%	—	Daily	—	Imminent brain death	Dc	High
Zeiler 2017^52^	Ns	64	54.2	100%	0%	—	PA, R	adm	10.3	Mortality, GOS	1mo, 6mo	Mod

Setting^a^: N/S, no specific location; Ns, neurosurgical unit; ED, emergency department; MG.ICU, medical/general intensive care unit; N.ICU, neurological intensive care unit; M.W, medical ward; M.HDU, medical high dependency unit; CCU, critical care medicine unit; N, neurological unit; S.ICU, surgical intensive care unit; ABIC, ambulatory brain injury clinic; Ns.ICU, neurosurgical intensive care unit.

Causes^b^: causes of deterioration in LOC. Neurological causes cover all primary neurological conditions, including trauma and anoxic-ischaemic encephalopathy. Non-neurological causes include metabolic encephalopathy, sepsis, categorized under other systems, and unknown or uncategorized causes.

Patient intubated^c^: 0% if the study excluded these patients.

Observer^d^: P, physician(s); PA, physician assistant(s); N, nurse(s); R, resident(s); NPsy, neuropsychologist(s); Sp, specialist(s); Ne, neurologist(s); NIn, neurointensivist(s); Iv, investigator(s); SHO, senior house officer(s); F, fellow(s); Con, consultant(s); In, intensivist(s); NsR, neurosurgery resident(s); Stu, student(s).

Assessment timing from admission^e^: adm, on admission; min, minute(s); hr, hour(s); d, day(s); mo, month(s); Dc, on discharge; post-sed, post-sedation.

Outcome^f^: only reports validated outcomes. GOS, Glasgow Outcome Scale; mRS, modified Rankin Scale; CPC, Cerebral Performance Categories; extub, extubation; rec, recovery.

Outcome timing^g^: Dc, on discharge or in-hospital death; d, day(s); mo, month(s).

Overall RoB: overall risk of bias for the study.

med^*^: median age was reported in place of mean.

^†^ These three studies are considered to be formed of the same study population.

^¶^ Total more than 100% because each patient could have multiple causes of DOC.

^‡^ The 2016 study is a follow-up of the same cohort reported in 2014.

^§^ The observers have never worked in neuroscience ICU or received formal neuroscience training.

In six studies, <50% of patients had an impairment of consciousness attributable to a neurological cause.^15,30–34^ Fourteen reports included solely patients with TBI.^16,17,39–42,19,25–27,35–38^ Twenty other studies included patients with non-traumatic neurological causes of impairment of consciousness, including brain tumor, intracranial aneurysm, stroke, encephalopathy, seizure, pneumocephalus, and hydrocephalus.^7,14,31,34,43–50,15,51,52,20–23,28–30^ Three studies excluded patients who were intubated,^14,38,45^ whereas 12 studies included mostly (>50% of study population) intubated patients.^19,25,48,51,30,32,34,35,40,41,43,44^

All studies, except one by Rohaut and colleagues,^34^ included study population with FOUR score ranging from 0–3 to 16 and mean total FOUR score between 4^34^ and 14.^50^

### Quality appraisal

Of the 40 records, only two achieved overall low risk of bias, 25 have moderate risk of bias, and 13 have high risk of bias (Table 3). The overview of the risk of bias of records differentiated by the reported outcome is shown below (Table 4).

**Table 3.** Quality Assessment for Included Records




PF, prognostic factor.

**Table 4.** Overview of Risk of Bias of Studies for each Outcome Reported by the Respective Studies

		*Number of studies with overall risk of bias of:*		
		*Low*	*Moderate*	*High*
Mor	<15-day/in-hospital	1	16	12
	≥15-day	2	7	2
GOS/GOSE	<3 months/at discharge	0	4	1
	3 to 6 months	0	5	0
	>6 months	0	1	0
mRS	<3 months/at discharge	0	4	0
	3 to 6 months	1	8	0
	>6 months	0	0	0
Others	Cerebral Performance Categories	0	2	0
	Intubation	0	1	0
	Extubation failure	0	1	0
	Awareness recovery	0	1	0
	Imminent brain death	0	0	1

Mor, mortality; GOS, Glasgow Outcome Scale; GOSE, extended Glasgow Outcome Scale; mRS, modified Rankin Scale.

Note: Total number exceeds 40 because some studies reported multiple outcomes or timings of outcome assessment.

### Mortality

Among the included studies, 36 measured mortality as the primary outcome. Of these studies, 25 described in-hospital mortality^7,14,23,24,29,33,35,36,38–41,15,42,45–47,49,16–22^ as the end point, 5 investigated mortality only up to 2 weeks post-event,^25,26,28,37,38^ and 11 studies explored longer-term mortality up to 3–6 months.^14,25,52,26,30–32,34,42,44,45^ The mortality rate between included studies varied widely—from 7.8%^36^ to 70%^28^—among studies where FOUR score was assessed within 24 h of admission. Studies using later time points of FOUR score assessment reported mortality within the above range (Supplementary Table S3). The highly variable mortality rate is likely a consequence of wide study methodological heterogeneity and thus no statistical association of timing of assessment and mortality was tested for.

#### Area under the curve

Of the same 36 studies reporting mortality, 30 reported the discriminative ability of FOUR score in predicting mortality by AUC.

##### a) Short-term (up to 2 weeks) and in-hospital mortality

**FIG. 2.**
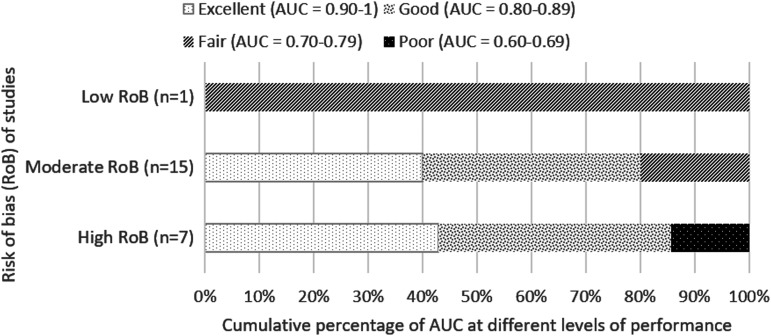
Cumulative percentage of AUC at different levels of performance (from poor to excellent) differentiated by overall risk of bias of studies for predicting in-hospital and short-term mortality. AUC value calculated based on first recorded total FOUR score. AUC, area under the curve; FOUR, Full Outline of UnResponsiveness.

Twenty-three studies reported short-term and in-hospital mortality, with 16 studies having found FOUR score to be good or excellent (AUC value, >0.80) in predicting in-hospital and short-term mortality,^7,16,29,35–37,39–41,46,17–21,23,25,26^ six of which have high risk of bias whereas the remaining are of moderate risk (Fig. 2). Both Kocak 2012^28^ and Mansour 2015^22^ found that for in-hospital and short-term mortality, FOUR score assessed at 3 days post-admission has higher predictive value (AUC >0.90) compared to assessment in the first day of admission (AUC <0.80), with non-overlapping confidence intervals which may suggest significant difference.

##### b) Long-term mortality (beyond 2 weeks)

**FIG. 3.**
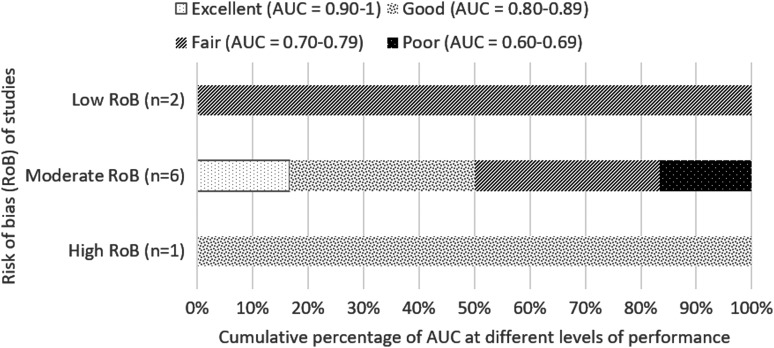
Cumulative percentage of AUC at different levels of performance (from poor to excellent) differentiated by overall risk of bias of studies for predicting long-term mortality. AUC value calculated based on first recorded total FOUR score. Zeiler 2017, a moderate-quality study, assessed mortality at 1 and 6 months, and both AUC values are included in this figure. AUC, area under the curve; FOUR, Full Outline of UnResponsiveness.

For longer-term mortality (beyond 2 weeks), FOUR score was determined to be mostly fair or good (AUC value, 0.70–0.89), as shown in eight studies,^14,25,26,30,31,34,44,50,52^ two of which have low risk of bias^14,34^ (Fig. 3). Zeiler 2017, a moderate risk of bias study, analyzed both 1- and 6-months mortality (AUC 0.76 and 0.82, respectively), both of which are included in Figure 3. The study by Weiss and colleagues^32^ was excluded from Figure 3 because the AUC value was based on differences between FOUR scores obtained on day 3 and day 1, so it was not possible to determine a discrete value for day 1.

##### c) Comparison with Glasgow Coma Scale

For studies which calculated the AUC value for GCS with the same assessment and outcome timing as FOUR score, the AUC value ranged from 0.62^28^ to 0.99,^50^ with overlapping confidence intervals with the corresponding AUC values for FOUR score, which may suggest a lack of significant difference between the scores. Among the 8 studies^7,36,37,39,40,45,46,52^ which did not state confidence intervals for AUC values, the difference in AUC value between GCS and FOUR score ranges from no difference in Wijdicks 2005^7^, to AUC value 0.064 lower in GCS than that of FOUR in study by Babu and colleagues.^40^

##### d) Performance of each Full Outline of UnResponsiveness score component

**FIG. 4.**
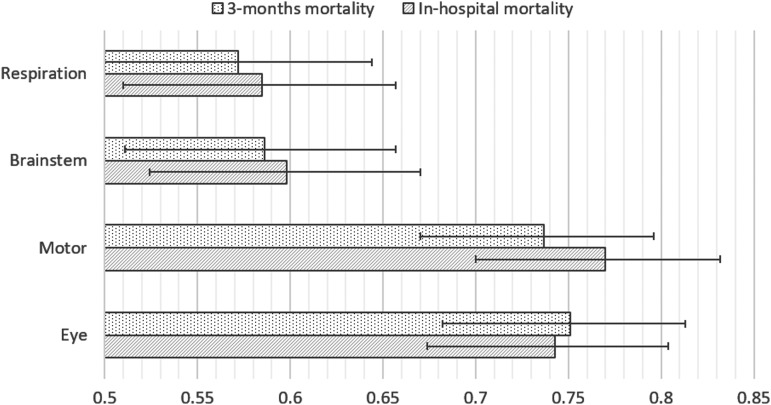
AUC values of different FOUR score components in predicting mortality in Eken 2009. AUC values calculated based on FOUR score assessed on admission. AUC, area under the curve; FOUR, Full Outline of UnResponsiveness.

**FIG. 5.**
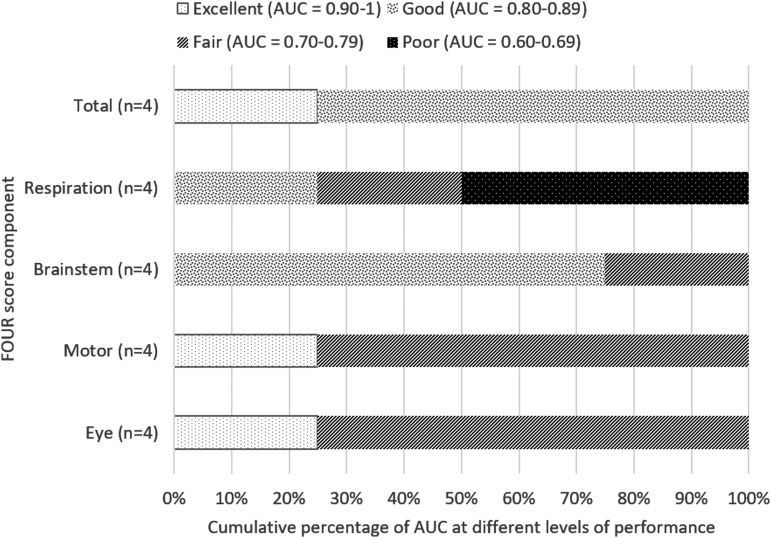
Cumulative percentage of AUC at different levels of performance. AUC values calculated based on day 1 FOUR score in predicting in-hospital mortality among studies of moderate risk of bias (Marcati 2012, Momenyan 2017, Okasha 2014, Peng 2015). AUC, area under the curve; FOUR, Full Outline of UnResponsiveness.

Eight studies reported AUC values for each component of FOUR score.^14,16,19,23,29,41,44,52^ Among those, only Eken 2009^14^ has low risk of bias. Eken and colleagues recruited 185 patients and demonstrated lower predictive performance for both respiration and brainstem components of FOUR score compared to motor and eye components (Fig. 4). Another four studies^19,23,29,41^ of moderate risk of bias with total recruitment of 351 patients showed mixed results for different components, with motor component having excellent prognostic performance only in Marcati 2012^29^ (Fig. 5). Gorji 2014,^16^ a study with high risk of bias, showed excellent predictive performance (AUC, >0.9) for motor and brainstem components. The remaining studies of moderate risk of bias, Chen 2013^44^ and Zeiler 2017,^52^ were not included in this analysis because mortality was assessed at 30 days, whereas all the other six studies measured in-hospital mortality; these studies have similar performance between different components.

#### Logistic regression

Seventeen studies used logistic regression to model dependence of survival on FOUR score^7,14,37,41,42,45–47,49,16,17,19,21,22,24,35,36^; one with overall low risk of bias^14^ and five studies are deemed high risk,^16,17,21,37,42^ whereas the 11 remaining studies have moderate risk of bias. These studies identified that for every 1-point increase in total FOUR score, the unadjusted odds ratio of in-hospital mortality ranged between 0.93^42^ and 0.59,^19^ indicating reduction in odds of mortality by 7% to 41% per 1-point increase in FOUR score. Two studies utilized 3-month mortality as the dependent variable and demonstrated odds of 3-month mortality per increase in total FOUR score of 0.69 in one unadjusted model^45^ and 0.64 in another model after adjustment for age, sex, blood pressures, respiration rate, alcohol, hypoglycemia, and trauma.^14^

### Glasgow Outcome Scale

Nine studies evaluated the GOS as a dichotomous index at various time points, ranging from discharge,^16,20^ 1 month,^19,44,52^ 3–6 months,^27,35,36,43,52^ and 12 months.^27^ Among these studies, only Gorji 2014^16^ has high risk of bias, whereas the other studies have moderate risk of bias. The percentage of the study population with poor outcome (GOS 1–3) ranged from 23.4%^27^ to 43.3%^35^ when assessed within 3 months from injury. The results of individual studies are shown in Supplementary Table S4.

#### Area under the curve

**FIG. 6.**
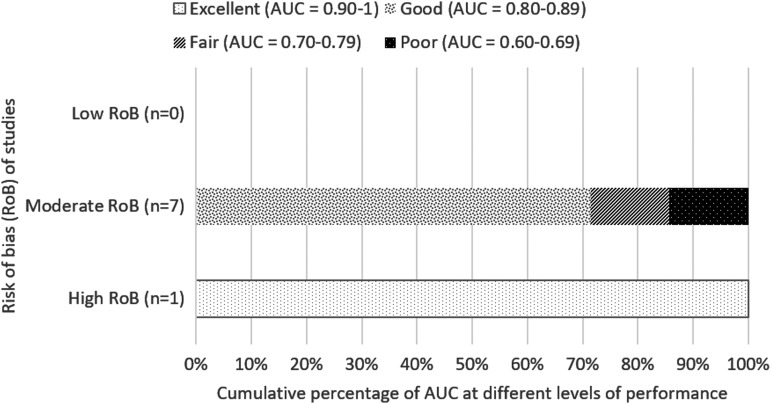
Cumulative percentage of AUC at different levels of performance (from poor to excellent) differentiated by overall risk of bias of studies for predicting poor outcome (GOS 1–3 or GOSE 1–4). AUC value calculated based on first recorded total FOUR score. Akavipat 2011 has been excluded from the figure as the AUC value is calculated based on GOS 3–5. AUC, area under the curve; FOUR, Full Outline of UnResponsiveness; GOS, Glasgow Outcome Score; GOSE, Glasgow Outcome Score–Extended.

All nine studies described the ability of FOUR score to predict poor outcome with AUC, which was found to be good (AUC value, 0.80–0.89) in five studies^19,27,35,36,52^ (Fig. 6). Akavipat 2011^20^ has been excluded from Figure 6 because the AUC value corresponds to GOS 3–5. The AUC values for GCS of the same timing for assessment and outcome as FOUR score ranged from 0.68^43^ to 0.90,^16^ which is similar to the corresponding AUC for FOUR score as shown in Supplementary Table S4.

#### Logistic regression

Five of the nine studies analyzed the odds ratio of poor outcome for cumulative increase in total FOUR score.^16,19,35,36,43^ The studies differed methodologically. It is likely that there is no statistically significant difference between these five different study groups in terms of odds ratio of poor outcome because the 95% confidence intervals were overlapping. Unfortunately, *p* values were only reported in two of these studies,^19,43^ and so it is not possible to ascertain what value of statistical significance was met. In the strong study by Kasprowicz and colleagues, poor outcome at 3 months was more strongly inversely associated with discharge (Expβ, 0.487) than admission (Expβ, 0.765) cumulative FOUR score, by logistic regression.

#### Relation to mortality

Among studies which investigated both GOS and mortality,^16,19,20,27,35,36,44,52^ the AUC for GOS is lower in seven^19,20,27,35,36,44,52^ of the eight studies; and where the confidence interval was reported, they overlapped with that for mortality in the same study, thus likely representing no significant difference.

### Modified Rankin Scale

Fourteen studies assessed mRS as the outcome at different time points—upon discharge,^29,41,47^ 1 month,^24,52^ and 3–6 months.^7,14,22,23,30,36,45,46,50,52^ The percentage of study population with poor outcome (mRS, 3–6) ranged from 29.4%^36^ to 76%.^24^ The results of each study are available in Supplementary Table S5. Zeiler 2017^52^ is not included in this table because the author only reported the association of FOUR score with mRS in exponent values, which is not comparable to other studies which calculated the AUC or odds ratio. Zeiler and colleagues found a statistically significant association between admission total FOUR score with 1-month (Exp, 0.609; *p* = < 0.001) and 6-month (Exp, 0.757; *p* = < 0.001) poor mRS outcome; significant association between day 7 total FOUR score and 6-month poor mRS outcome (Exp, 0.469; *p* = 0.009); whereas day 14 total FOUR score does not have statistically significant association with poor outcome (Exp, 0; *p* = 0.992 for 1-month poor outcome; Exp, 0.199; *p* = 0.165 for 6-month poor outcome).

#### Area under the curve

**FIG. 7.**
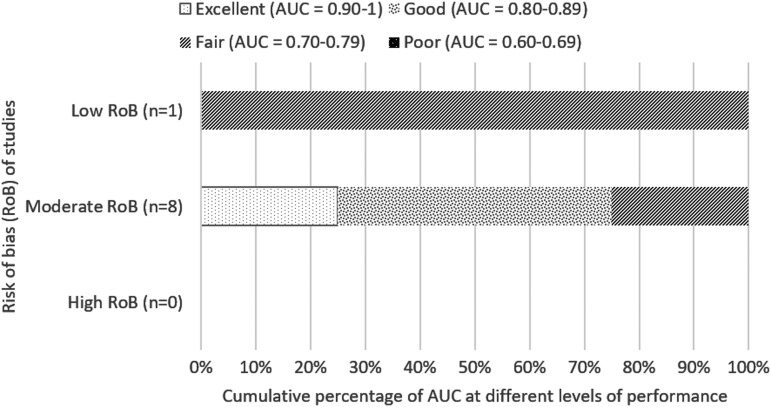
Cumulative percentage of AUC at different levels of performance (from poor to excellent) differentiated by overall risk of bias of studies for predicting poor outcome (mRS 3–6). AUC value calculated based on first recorded total FOUR score. AUC, area under the curve; FOUR, Full Outline of UnResponsiveness; mRS, modified Rankin Scale.

Eleven of the 14 studies analyzed performance by determining the AUC value for the ability of total FOUR score to predict poor mRS outcome. This was generally considered fair or good (AUC, 0.70–0.89) as shown in Figure 7. Surabenjawong 2017 and Gujjar 2013 are not included in Figure 7 because of different cut-off values of mRS used in their studies (4–6 and 0–3, respectively). The AUC values for GCS of the same assessment time point and outcome as their FOUR score counterpart ranged from 0.68^45^ to 0.99,^41^ as shown in Supplementary Table S5.

#### Logistic regression

The odds ratio of poor outcome at 3 months for every 1-point increase in day 1 total FOUR calculated by the seven studies which reported it^7,22,24,36,41,46,47^ ranged between 0.84^7^ and 0.15.^41^

### Other outcomes

Other outcomes, each reported only in one study,^19,30,32,48,50,51^ are summarized in Table 5.

**Table 5.** Other Reported Outcomes in the Studies Included

		*Outcome*										
	*FOUR timing*	*Time*	*Outcome*	*Pt, %*	*AUC (95% CI)*	*Cut off*	*Sn, %*	*Sp, %*	*PPV, %*	*NPV, %*	*GCS AUC (95% CI)*	*Risk of bias*
Weiss 2015	Δ3d–1d 3d	6mo	CPC 3–5	77.6	0.87^a^ (0.74–0.94) — — —	— 4 8 10	— 80 96 100	— 65 53 53	— 86 84 85	— 55 82 100	0.75^a^ (0.56–0.86) — — —	Mod
Surabenjawong 2017	adm	3mo	CPC 3–5	32	1.00 (1.00–1.00)	10	—	—	—	—	0.94 (0.91–1.02)	Mod
Okasha 2014	adm	—	intub at ER	78.3	0.961	11	79	100	—	—	0.982	Mod
Said 2016	0–24hr of intub 14d	14d	extub failure	69.8	0.867^*^ (0.790–0.944) 0.95 (0.90–0.99)	10 12	80.8 92.3	81.7 85.0	— —	— —	0.832^*^ (0.741–0.923) 0.71 (0.60–0.82)	Mod
Hu 2017	3d	90d	awareness recovery	60	0.819 (0.723–0.883)	—	—	—	—	—	0.875 (0.795–0.932)	Mod
Zappa 2017	daily	Dc	imminent brain death	65	—	—	100	53.8	53.8	100	—	High

Outcome: time, timing of outcome assessment; CPC, cerebral performance categories; intub at ER, intubation at emergency room; extub, extubation;

Timing: adm, on admission; hr, hour(s); d, day(s); mo, month(s); DC, on discharge.

Risk of bias: Mod, moderate.

^a^ Value based on delta day 3–day 1 (i.e., difference in score between day 3 and day 1).

^*^ Significant difference between FOUR and GCS, *p* = 0.014.

FOUR timing, timing of FOUR score assessment relative to the injury date, unless stated otherwise; Pt, percentage of study population achieving the outcome; AUC, area under receiver operating characteristic curve; Cut off, cut-off value of FOUR score for logistic regression; Sn, sensitivity; Sp, specificity; PPV, positive predictive value; NPV, negative predictive value; CI, confidence interval; SD, standard deviation.

### Subgroup comparisons

#### 1) Between patients with neurological and non-neurological causes of impaired consciousness

A total of 2359 patients were recruited in the group with neurological causes of impaired consciousness, of which 1174 were from 14 studies which only included patients with TBI; whereas 2367 patients were recruited in studies of predominantly non-neurological causes of impaired consciousness (Supplementary Table S6). Studies of both groups differed widely in time point for outcome assessment, precluding meta-analysis. Among studies which reported the 95% confidence interval for AUC, the confidence intervals of four^19,22,23,49^ of the six studies with purely neurological causes of impaired consciousness overlap with the confidence interval of the study with the same time point and outcome assessment (in-hospital mortality) in the non-neurological group^15^; the remaining two^16,17^ of the six studies have their confidence intervals overlapping with that of all studies of the same outcome assessment in the neurological group. Therefore, comparison between patients with neurological and non-neurological causes of impaired consciousness was inconclusive because of heterogeneity between studies. Among studies of purely neurological cause of impaired consciousness which assessed in-hospital mortality, the AUC value ranges between 0.76^49^ and 0.93^36^; whereas the AUC value ranges between 0.70^15^ and 0.84^30^ among studies of predominantly non-neurological cause.

#### 2) Between patients in specialized neurological units and non-neurological units

Only five studies were done in a neurological ICU with total recruitment of 474 patients, whereas 3568 patients were included in 19 studies performed in a non-neurological ICU. Different studies recruited observers of different backgrounds to assess FOUR score, ranging from nurses to neurologists. Both groups of studies have similar and overlapping AUC values between 0.7 and 0.9, suggesting no difference in performance between the units (Supplementary Table S7). Only one study of patients from a neurological ICU reported 95% confidence intervals, and these overlap with all three^15–17^ studies of patients from non-neurological ICUs of the same time point and outcome assessment (in-hospital mortality), which reported 95% confidence intervals. Marcati 2012^29^ and Babu 2017^40^ were excluded from this analysis because of inclusion of patients from both neurological and non-neurological units.

#### 3) Between intubated and non-intubated patients

Twelve studies included mostly (>50% sample size) patients who were intubated, but each study had different outcome or time point of outcome (Supplementary Table S8). Only one study^14^ with no intubated patients in the study population reported the AUC with confidence interval, which overlaps with the AUC confidence intervals of two studies^19,41^ with the majority of intubated patients of the same time point and outcome assessment (in-hospital mortality). The studies were too heterogeneous to form a conclusion as to whether FOUR score was of more value in one patient group or the other.

## Discussion

We have demonstrated that FOUR score overall has a close relationship to in-hospital mortality and poor functional outcome in patients with impaired consciousness. Our study identified 40 records for inclusion, but the significant differences between the studies with regard to the characteristics of patient population, time points of assessment and outcome, and characteristics of observer precluded direct comparison in a meta-analysis. The studies had mixed methodological qualities.

The only previous systematic review studying FOUR score's ability to predict outcomes was performed by Seel and colleagues in 2010.^53^ That study included four studies only,^7,14,24,47^ most of which had high or very high risk of bias. The authors were unable to make a confident conclusion as to the prognostic validity of FOUR score.^53^ The strengths of our review are that the search strategy was comprehensive to cover all studies related to FOUR score, including gray literature, and the review was done according to the accepted best reporting practice^8^ for systematic review. Duplicate work by another author reduced risk of bias. However, there were limitations in this review. Non-English articles were excluded from review. It is possible that additional information, including individual patient-level data, could have been retrieved by contacting the authors of included studies to permit meta-analysis. However, by ensuring rigorous adherence to relevant STROBE guidelines,^54^ in reporting of such studies, authors and journal editors can ensure that published data contribute meaningfully to future meta-analyses. Unfortunately, the highly heterogeneous nature of included study populations, study design, and study reporting necessarily limit the strength of our conclusions. Twenty-nine of the included studies^14,16,30,32–34,36–41,19,42–46,48,49,51,52,21–25,28,29^ did not calculate the power of the sample size in their studies. There was considerable variation between the studies in the pathologies of the patient population, the location of treatment, and whether they were intubated.

The FOUR score had good or excellent relationship to in-hospital mortality in 16 studies^7,16,35,36,39–41,46,17–23,29^ included in this review. It has good performance relating to in-hospital mortality and poor functional outcome in the acute setting involving general ICU or ED populations presenting with intracranial lesions and cardiac arrest.

The statistical method chosen by most studies to measure the prognostic ability of FOUR score was by calculating its AUC in relation to the outcome of interest. AUC allows comparison of test performance between different tests,^55^ and these studies^7,14,25–32,35,36,15,37,39–41,43–46,49,50,16,52,17–19,21–23^ used AUC to quantify the performance of FOUR score in predicting mortality or poor functional outcome, compared to the performance of the GCS. However, three of the studies^28,31,50^ only calculated the AUC without providing additional statistical analyses, such as its sensitivity, specificity, or odds ratio, thus limiting the usefulness of the result because the AUC value could only be used to compare with another tool, but not to aid clinical decision-making itself.^56^ For instance, both Akavipat and colleagues^20^ and Gorji and colleagues^16^ found that FOUR score has an AUC value of 0.92 for predicting in-hospital mortality, but the sensitivity was only 58% at FOUR score value of 8 in the former study,^20^ whereas the sensitivity was 90% at FOUR score value of 4 in the latter.^16^ Hence, additional analyses of the raw study data, such as the odds ratio of adverse outcome for every point increase in FOUR score, would help in making clinical decisions. There may also be merits in reporting FOUR score components separately in clinical care, as for GCS.^2,3^

Despite the overall agreement between studies that FOUR score can make a prediction about mortality and poor functional outcome, wide variation of the AUC for different outcomes exists between studies. The differences may be influenced by factors such as different study populations, variation in the time points of assessment, and variation in competency of the practitioner assessing level of consciousness using FOUR score. The quality appraisal process has also identified 11 studies^16,18,51,21,25,26,33,37,38,40,42^ with high risk of bias caused by confounders which were inadequately controlled, which could explain the variation in AUC values compared to studies of lower risk of bias. There were differences in AUC values between mortality and poor outcome measured by GOS within the same studies; the AUC value for GOS was generally lower than that of mortality, but the difference may not be significant as indicated by overlapping confidence intervals. The lower AUC value for GOS may be a result of longer follow-up period or misclassification with GOS.^57^ It was unclear in the studies exactly what added value a cut-off level had for implementation in clinical practice. Addressing this might help standardize the methodological approaches.

Three studies, two^18,28^ of which have high risk of bias and one^22^ with moderate risk of bias, which analyzed the ability of FOUR score to predict in-hospital mortality with different time points of assessment of FOUR score, showed greater AUC values when FOUR score is assessed later post-admission compared to the first assessment performed in each study. These studies have excluded patients who were sedated. Kocak and colleagues^28^ and Mansour and colleagues.^22^ included stroke patients in their studies and found higher AUC value with FOUR score assessed at day 3 post-admission compared to day 1, with non-overlapping confidence interval. This may suggest that prognostic ability of FOUR score may improve with time as the nervous system recovers from the initial insult. However, these studies consist of heterogenous population, with 70% mortality rate in Kocak 2012 but 20% mortality rate in Mansour 2015, and with different time points of outcome assessment, the strength of a pooled result would be limited, while lack of access to raw data restricted our ability to perform further statistical analysis to determine significance. Therefore, future studies should consider investigating the relation of timing of FOUR score assessment and outcome with statistical analysis. The difference in timing of assessment of FOUR score between studies may affect the results in each study. Given that 28 of the included studies assessed FOUR score within the first day of admission, the overall prognostic ability measured by AUC may be better had the studies performed the assessment later.

Many of the studies reviewed identified similar performance between FOUR score and GCS,^14,17,35,37,43,18,22,23,27–31^ as shown in Supplementary Tables S3–Supplementary Tables S5. Given that the comparison of performance between FOUR score and GCS is not the primary aim of this review, it may be beneficial to have a further study comparing the prognostic ability of both scores through a new review strategy, including subgroup analysis of certain patient groups such as those with TBI and the performance of each component in both scores. However, individual components of FOUR score which were supposed to address the shortcomings of GCS, namely brainstem and respiratory pattern,^7^ showed significantly poorer performance than eye and motor components of FOUR score in a methodologically strong study with low risk of bias by Eken and colleagues.^14^ This study also included patients who did not have impaired level of consciousness upon presentation to an emergency department. Consequently, the poorer performance of brainstem and respiratory pattern components may partly be attributed to the relatively neurologically well study population. This is perhaps in keeping with Wijdicks and colleagues' claim that FOUR score performs better than GCS at lower levels of consciousness. The variation in prognostic performance between components may be a result of floor and ceiling effects of the components as observed in the GCS,^3^ with each component contributing differentially across the spectrum of consciousness. Whereas eye and motor components of the GCS represent the global impact of the neurotrauma on the brain, the brainstem and respiratory pattern components of FOUR score are perhaps assessing more-specific brainstem injury, hence the variation seen between patients with different severities of TBI.

The other study of overall low risk of bias by Rohaut and colleagues^34^ only included deeply sedated patients who were mechanically ventilated and excluded patients with TBI or other neurological disorders. Here, an AUC of 0.76 was reported for prediction of 28-day mortality using total FOUR score, suggesting that FOUR score does not necessarily perform better in patients with lower consciousness. In a general TBI population commonly seen in the emergency department, the prognostic ability of FOUR score is likely similar to the performance determined by Eken and colleagues (AUC, 0.78–0.79 for in-hospital and 3-month mortality) given that the study includes general TBI patients with normal consciousness on admission. The demonstration of similar predictive values of FOUR score in two studies of low risk of bias using different populations and in different settings provides some confidence as to the generalizability of these findings.

A similar finding is reported by Marcati and colleagues,^29^ which has an overall moderate risk of bias, where the brainstem and respiratory pattern components have lower AUC values. Momenyan and colleagues^41^ found that brainstem and respiratory pattern components performed better than eye and motor components, but did not demonstrate statistical significance. Wijdicks and colleagues^15^ reported that these components contribute to improved prognostication of FOUR score, but does not appear to have reported data to justify this.

The eye and motor components of FOUR score are not the same as those in the GCS. It is unclear whether a specific subpopulation of patients with reduced consciousness would benefit most from the FOUR score compared to GCS. The available evidence did not allow determination of the prognostic contribution of brainstem components of FOUR score in addition to those already present in the GCS. The recently described GCS-Pupils (GCS-P) score demonstrated that the simple addition of pupil reactivity information to the GCS increased the amount of variation in patient outcome explained by the model, as assessed with Nagelkerke's *R*^2^ test.^58^

A lack of clarity about whether observers were trained in using FOUR score could have influenced the results of the studies. The added complexity of assessing brainstem function compared to the eye, verbal, and motor components of the GCS may contribute to variations in scoring, particularly outside a research study. The study by Gujjar and colleagues^45^ recruited neurology consultants who were briefed in the use of FOUR score, whereas observers in study by Wijdicks and colleagues^15^ received comprehensive training delivered by experienced critical care nurse and an educational video. Other studies did not report who performed the assessment or whether observers received training of FOUR score assessment beforehand.^21,22^ Training has the single most clear effect on the reliability of GCS.^59^ Therefore, development of a structured education tool for FOUR score, similar to the online GCS education tool^60^ (www.glasgowcomascale.org), could improve its reliability and reduce variability of FOUR score assessment of level of consciousness, which is crucial if FOUR score is to be used clinically outside of a research setting.

## Conclusion

FOUR score overall has a close relationship to in-hospital mortality and poor functional outcome in patients with impaired consciousness. There was insufficient evidence to determine whether performance differs in different groups. There was some suggestion that assessment of brainstem reflexes and respiratory pattern made less contribution than eye and motor scores. Future studies would benefit from standardizing research methodology. They should include larger populations with adequate power, preferably with stricter inclusion criteria including standardized timing of assessment in relation to injury, and regarding the pathology causing the reduced consciousness. Further comparison of FOUR score and GCS, and with GCS-P, may, in subgroups of patients, identify relative merits of FOUR score.

## Supplementary Material

Supplemental data

Supplemental data

Supplemental data

Supplemental data

Supplemental data

Supplemental data

Supplemental data

Supplemental data
