# Supplementary material for: The Relationship of the FOUR Score to Patient Outcome: A Systematic Review
Source: J Neurotrauma. 2019 Aug 20;36(17):2469–83. doi: 10.1089/neu.2018.6243 (PMC6709730; doi:10.1089/neu.2018.6243)
Supplement: Supplemental data [file Supp_Table1.pdf]

MEDLINE (PubMed) :
"four score"[All Fields] OR "full outline of unresponsiveness"[All Fields] AND ("2005/01/01"[PDAT] : "2018/04/30"[PDAT])

The Cochrane Central Register of Controlled Trials (CENTRAL) :

ID	Search
#1	"four score":ti,ab,kw (Word variations have been searched)
#2	"full outline of unresponsiveness":ti,ab,kw (Word variations have been searched)
#3	#1 or #2 Publication Year from 2005 to 2018

EMBASE

1	("four score" or "full outline of unresponsiveness").ab,ti.
2	limit 1 to yr="2005 - 2018"

Scopus
TITLE-ABS ("four score" OR "full outline of unresponsiveness") AND PUBYEAR > 2004 AND PUBYEAR < 2019

Web of Science
TS=("four score" OR "full outline of unresponsiveness")
Indexes=SCI-EXPANDED, SSCI, A&HCI, CPCI-S, CPCI-SSH, BKCI-S, BKCI-SSH, ESCI, CCR-EXPANDED, IC Timespan=2005-2018

ClinicalTrials.gov
"four score" OR "full outline of unresponsiveness"

OpenGrey
"four score" OR "full outline of unresponsiveness"

Supplementary Table S1. Search strategy
