# Supplementary material for: The Relationship of the FOUR Score to Patient Outcome: A Systematic Review
Source: J Neurotrauma. 2019 Aug 20;36(17):2469–83. doi: 10.1089/neu.2018.6243 (PMC6709730; doi:10.1089/neu.2018.6243)
Supplement: Supplemental data [file Supp_Table2.pdf]

Study :<br>Name of author(s);<br>Year of publication;<br>Country and setting where study was conducted;<br>Study design;<br>Observer;<br>Inclusion and exclusion criteria;<br><br>Population :<br>Sample size;<br>Is there a sample size calculation (Y/N);<br>Age range;<br>Setting;<br>Cause of impairment;<br><br>Intervention :<br>Time point of assessment;<br>FOUR score (max., min., mean, SD);<br>Area under ROC curve for each outcome;<br>Other statistical analysis of outcome (e.g. sensitivity, specificity, odds ratio);<br><br>Comparator :<br>GCS (max., min., mean, SD);<br>Area under ROC curve for each outcome;<br>Other statistical analysis of outcome (e.g. sensitivity, specificity, odds ratio);<br><br>Outcome :<br>Mortality;<br>Other validated measures of outcome (e.g. modified Rankin Scale, Glasgow Outcome Scale);<br>Time point of outcome assessment;

Supplementary Table S2. Data extracted from included study
