# Supplementary material for: The Relationship of the FOUR Score to Patient Outcome: A Systematic Review
Source: J Neurotrauma. 2019 Aug 20;36(17):2469–83. doi: 10.1089/neu.2018.6243 (PMC6709730; doi:10.1089/neu.2018.6243)
Supplement: Supplemental data [file Supp_Table3.pdf]

	FOUR timing	Mort. timing	Pt, %	AUC (95% CI)	Cut-off	Sn, %	Sp, %	PPV, %	NPV, %	OR (95% CI)	GCS AUC (95% CI)	Risk of bias
**Akavipat 2011**	0-30min	in-hosp	12.5	0.92 (0.87-0.97)	8	58	93	54	94	-	-	Mod
					10	71	93	60	96	-		
**Babu 2017**	0-1d	in-hosp	30.6	0.860	8	79.9	81.6	90.3	60.5		0.796	High
**Baratloo 2016**	adm	in-hosp	32.9	0.86 (0.79–0.94)	-	-	-	-	-	-	0.85 (0.77-0.93)	High
	6h			0.93 (0.89-0.98)	-	-	-	-	-	-	0.93 (0.88-0.98)	
	12h			0.95 (0.91-0.99)	-	-	-	-	-	-	0.95 (0.90-1.0)	
**Chen 2013**	0-1d	30d	31.7	0.768 (0.664-0.872)	4	50	96	-	-	-	0.699 (0.595-0.802)	Mod
**Eken 2009**	adm	in-hosp	14.1	0.788 (0.722–0.844)	-	-	-	-	-	-	0.735 (0.655-0.797)	Low
		3mo	25.4	0.776 (0.709–0.834)	9	-	-	-	-	0.638* (0.511-0.796)	0.726 (0.656-0.789)	
**Fischer 2010**	0-3d	28d	13	0.79 (0.69-0.89)	-	-	-	-	-	-	0.78 (0.68-0.87)	Mod
**Fugate 2010**	3d	in-hosp	65	-	4	47.6	100	100	67.2	-	-	High
				-	8	90.5	91.1	90.5	91.1	-		
				-	10	92.9	80.0	81.3	92.3	-		
**Gorji 2014**	0-24hr	in-hosp	18.9	0.92 (0.81-0.97)	4	90	90	-	-	0.75	0.96 (0.87-0.99)	High
**Gorji 2015**	0-24hr	<14d	18.7	0.90 (0.88-0.90)	4	92	87	-	-	-	0.80 (0.78-0.84)	High
		>14d	7.5	0.86 (0.84-0.90)	6	90	82	-	-	-	0.89 (0.78-0.88)	
**Gujjar 2013**	0-24hr	in-hosp	24	-	-	-	-	-	-	0.759† (0.593-0.970)	-	Mod
		3mo	39	0.697†	-	-	-	-	-	0.694† (0.574-0.839)	0.655†	
**Hosseini 2017**	0-24hr	<14d	13.7	0.92 (0.81-0.97)	4	90	90	-	-	-	0.96 (0.87-0.99)	High
		>14d	5	0.89 (0.81-0.94)	6	73	90	-	-	-	0.90 (0.87-0.95)	
**Iyer 2009**	-	in-hosp	33	0.86	-	-	-	-	-	0.75 (0.68-0.84)	0.82	Mod
**Jalali 2014**	0-1d	in-hosp / ≤14d	36.5	-	6	68.4	77.3	63.4	81.0	-	-	High
**Kasprowicz 2016**	0-24hr	in-ICU	30.9	0.906‡ , SD=0.024	-	-	-	-	-	0.511* (0.382-0.684)	0.913‡, SD=0.022	Mod
**Khanal 2016**	0-24hr	in-ICU	29.9	0.82 (0.73-0.91)	6.5	79.3	79.4	62.2	90.0	0.70 (0.60-0.82)	0.79 (0.74-0.91)	High
**Kocak 2012**	1d	<15d	70	0.675 (0.565-0.786)	-	-	-	-	-	-	0.624 (0.510-0.738)	High
	3d			0.922 (0.867-0.977)	-	-	-	-	-	-	0.925 (0.873-0.977)	
	10d			0.981 (0.947-1.015)	-	-	-	-	-	-	0.982 (0.951-1.013)	
**Lee 2017**	0-1hr	in-hosp	12.4	0.758 (0.599-0.916)^{p}	-	-	-	-	-	0.756 (0.629-0.909)*^{p}	0.744 (0.579-0.910)^{p}	Mod
**Mansour 2015**	24h	in-hosp	19.7	0.796 (0.715-0.862)	11	84	57	-	-	0.64 (0.52-0.79)	0.779 (0.697-0.848)	Mod
	72h			0.977 (0.933-0.995)	8	100	86	-	-	0.26 (0.12-0.54)	0.975 (0.930-0.994)	
**Marcati 2012**	0-7d	in-hosp	25.3	0.935 (0.884-0.985)	10	91	86	-	-	-	0.953 (0.913-0.994)	Mod
**McNett 2014**	24hr	in-hosp	12.5	0.913 (0.822-1.00)	-	-	-	-	-	0.66 (0.56-0.77)	0.935 (0.876-0.995)	High
	72hr			0.837 (0.683-0.991)	-	-	-	-	-	0.76 (0.66-0.87)	0.884 (0.798-0.969)	
**Momenyan 2017**	0-7d	in-hosp	18.8	0.835 (0.739-0.907)	6	100	62	-	-	0.67 (0.54-0.85)	0.772 (0.668-0.856)	Mod

Supplementary Table S3. Results of studies investigating mortality as the outcome.

	FOUR timing	Mort. timing	Pt, %	AUC (95% CI)	Cut-off	Sn, %	Sp, %	PPV, %	NPV, %	OR (95% CI)	GCS AUC (95% CI)	Risk of bias
**Okasha 2014**	adm	in-hosp	25	0.850 (0.734-0.929)	9	73	80	-	-	0.59 (0.44-0.79)	0.796 (0.673-0.889)	Mod
**Peng 2015**	0-1d	in-hosp	21.7	0.834 (0.740-0.928)	9	75	85	-	-	-	0.815 (0.723-0.908)	Mod
**Rohaut 2017**	24hr post-sedation	28d	30	0.76 (0.67-0.84)[C]	-	-	-	-	-	-	-	Low
**Sadaka 2012**	0-24hr	in-hosp	7.8	0.93	-	-	-	-	-	0.64 (0.46-0.89)	0.89	Mod
**Said 2016**	≤24hr intub	28d	-	0.837 (0.748–0.926)	9	84.4	79.6	-	-	-	0.862 (0.785-0.939)	Mod
**Saika 2015**	adm	14d	12.3	0.97	7	97.5	88.2	-	-	0.348* (0.22-0.56)	0.95	High
**Senapathi 2017**	adm, 24hr, 48hr, 72hr	in-hosp	30.2	-	-	-	-	-	-	0.925 (0.858-0.996)	-	High
**Sepahvand 2016**	24-48hr	in-hosp	34.8	0.961	6	76	90	83	86	-	0.928	Mod
**Stead 2009**	-	in-hosp	-	-	-	-	-	-	-	0.67 (0.53-0.84)	-	Mod
**Surabenjawong 2017**	adm	3mo	16.7	1.00 (0.94-1.00)	10	-	-	-	-	-	0.99 (0.92-0.99)	Mod
**Weiss 2015**	Δ3d-1d	6mo	-	0.84[§] (0.69-0.92)	-	-	-	-	-	-	0.75[§] (0.58-0.86)	Mod
**Wijdicks 2005**	0-1d	in-hosp	21	0.81	9	75	76	-	-	0.80 (0.72-0.88)	0.81	Mod
**Wijdicks 2015**	0-1hr	in-hosp<br>in-ICU	11.6<br>7.8	0.702 (0.661-0.744)<br>0.742 (0.694-0.790)	-<br>-	-<br>-	-<br>-	-<br>-	-<br>-	-<br>-	0.684 (0.641-0.723)<br>0.715 (0.663-0.768)	Mod
**Wolf 2007**	0-24hr	in-hosp	29	-	-	-	-	-	-	0.62 (0.51-0.75)	-	Mod
**Zeiler 2017**	adm	1mo<br>6mo	-<br>≤15.6	0.762<br>0.823	-<br>-	-<br>-	-<br>-	-<br>-	-<br>-	-<br>-	0.753<br>0.820	Mod

Abbreviations: FOUR timing, timing of FOUR score assessment relative to the injury date unless stated otherwise; Mort. timing, timing of mortality assessment unless stated otherwise; Pt, percentage of study population achieving the outcome; AUC, area under receiver operating characteristics curve; Cut-off, cut-off value of FOUR score for logistic regression; Sn, sensitivity; Sp, specificity; PPV, positive predictive value; NPV, negative predictive value; OR, odds ratio in terms of every 1-point increase in FOUR score in relation to achieving the outcome of the study unless stated otherwise; CI, confidence interval; SD, standard deviation;

Timing: adm, on admission; min, minute(s); hr, hour(s); d, day(s); mo, month(s); in-hosp, in-hospital mortality; in-ICU, in-ICU mortality; intub, intubation;

GCS AUC: Assessment of GCS and outcome at the same time as FOUR score.

Risk of bias: Mod, moderate.

* - adjusted value (one or more of age, sex, aetiology, health status)

† - Score assessed by consultant

[p] - based on FOUR score assessed by physicians

‡ - integrated other significant predictors of outcome into the model for calculation

[C] – c-index value

§ - value based on delta day 3-day 1 (i.e. difference in score between day 3 and day 1)

Supplementary Table S3 (*continued*). Results of studies investigating mortality as the outcome.
