# Supplementary material for: The Relationship of the FOUR Score to Patient Outcome: A Systematic Review
Source: J Neurotrauma. 2019 Aug 20;36(17):2469–83. doi: 10.1089/neu.2018.6243 (PMC6709730; doi:10.1089/neu.2018.6243)
Supplement: Supplemental data [file Supp_Table4.pdf]

	FOUR timing	GOS		Pt, %	AUC (95% CI)	Cut-off	Sn, %	Sp, %	PPV, %	NPV, %	OR (95% CI)	GCS AUC (95% CI)	Risk of bias
		Time	Sc										
**Akavipat 2011**	0-30min	Dc	3-5[#]	40.1	0.88 (0.83-0.92)	14	77	95	90	86	-	-	Mod
**Bruno 2011**	0-1mo	3mo	1-3	-	0.70	-	-	-	-	-	0.83* (0.73-0.95)	0.68	Mod
**Chen 2013**	0-1d	30d	2-3	48.5	0.682 (0.531-0.832)	-	-	-	-	-	-	0.683 (0.533-0.832)	Mod
			4-5	18.8	0.748 (0.624-0.871)	-	-	-	-	-	-	0.727 (0.588-0.865)	
**Gorji 2014**	0-24hr	Dc	1-3	31.6	0.95 (0.86-0.99)	6	86	87	-	-	0.73	0.90 (0.79-0.96)	High
**Kasprowicz 2016**	0-24hr	3mo	1-3	43.3	0.852‡ SD=0.037	-	-	-	-	-	0.765* (0.654-0.884)	-	Mod
	Dc from ICU				0.895‡ SD=0.029	-	-	-	-	-	0.487* (0.315-0.754)	-	
**McNett 2016**	24h	6mo	1-3	23.4	0.813 (0.697-0.928)	-	-	-	-	-	-	0.818 (0.705-0.931)	Mod
		12mo		21.5	0.833 (0.718-0.948)	-	-	-	-	-	-	0.831 (0.713-0.949)	
	72h	6mo		23.4	0.738 (0.594-0.882)	-	-	-	-	-	-	0.764 (0.632-0.896)	
		12mo		21.5	0.758 (0.607-0.909)	-	-	-	-	-	-	0.796 (0.665-0.927)	
**Okasha 2014**	adm	1mo	1-4[¶]	58.3	0.813 (0.691-0.902)	11	80	64	-	-	0.54 (0.37-0.78)	0.779 (0.653-0.876)	Mod
**Sadaka 2012**	0-24hr	3-6 mo	1-3	29.4	0.85	-	-	-	-	-	0.67 (0.53-0.85)	0.83	Mod
**Zeiler 2017**	adm	1mo	1-3	-	0.810	-	-	-	-	-	-	0.796	Mod
		6mo			0.832	-	-	-	-	-	-	0.832	

Abbreviations: FOUR timing, timing of FOUR score assessment relative to the injury date unless stated otherwise; GOS, Glasgow Outcome Scale; Time, timing of GOS assessment unless stated otherwise; Sc, GOS score; Pt, percentage of study population achieving the outcome; AUC, area under receiver operating characteristics curve; Cut-off, cut-off value of FOUR score for logistic regression; Sn, sensitivity; Sp, specificity; PPV, positive predictive value; NPV, negative predictive value; OR, odds ratio in terms of every 1-point increase in FOUR score in relation to achieving the outcome of the study unless stated otherwise; CI, confidence interval; SD, standard deviation;

GCS AUC: Assessment of GCS and outcome at the same time as FOUR score.

Timing: adm, on admission; min, minute(s); hr, hour(s); d, day(s); mo, month(s); Dc, discharge;

Risk of bias: Mod, moderate.

* - adjusted value (one or more of age, sex, aetiology, health status)

‡ - integrated other significant predictors of outcome into the model for calculation

¶ - extended Glasgow Outcome Scale

# - author defined that as poor outcome

Supplementary Table S4. Results of studies investigating GOS as the outcome.
