# Supplementary material for: The Relationship of the FOUR Score to Patient Outcome: A Systematic Review
Source: J Neurotrauma. 2019 Aug 20;36(17):2469–83. doi: 10.1089/neu.2018.6243 (PMC6709730; doi:10.1089/neu.2018.6243)
Supplement: Supplemental data [file Supp_Table5.pdf]

	FOUR timing	mRS		Pt, %	AUC (95% CI)	Cut-off	Sn, %	Sp, %	PPV, %	NPV, %	OR (95% CI)	GCS AUC (95% CI)	Risk of bias
		Time	Sc										
**Eken 2009**	adm	3mo	3-6	38.8	0.751 (0.682-0.812)	-	-	-	-	-	-	0.720 (0.650-0.784)	Low
**Gujjar 2013**	0-24hr	3mo	0-3	32	0.708†	-	-	-	-	-	-	0.683†	Mod
**Iyer 2009**	-	3mo	3-6	66	0.75	-	-	-	-	-	0.82 (0.74-0.93)	0.76	Mod
**Mansour 2015**	24h<br>72h	3mo	3-6	56.7	0.865 (0.794-0.920)<br>0.909 (0.844-0.953)	11<br>11	79<br>84	85<br>94	-<br>-	-<br>-	0.55 (0.45-0.67)<br>0.53 (0.43-0.65)	0.868 (0.797-0.922)<br>0.931 (0.872-0.969)	Mod
**Marcati 2012**	0-7d	Dc	3-6	55.2	0.909	-	-	-	-	-	-	0.958	Mod
**Momenyan 2017**	0-7d	Dc	3-6	47.1	0.983 (0.928-0.999)	6	100	91.1	-	-	0.15 (0.04-0.6)	0.987 (0.934-1.000)	Mod
**Peng 2015**	0-1d	3mo	3-6	65	0.818 (0.742-0.894)	13	79	74	-	-	-	0.812 (0.734-0.891)	Mod
**Sadaka 2012**	0-24hr	3-6 mo	3-6	29.4	0.80	-	-	-	-	-	0.71 (0.57-0.88)	0.78	Mod
**Said 2016**	0-24hr of intub	3mo	3-6	-	0.897 (0.833-0.961)	9	100	72.7	-	-	-	0.907 (0.847-0.967)	Mod
**Stead 2009**	-	Dc	3-6	-	-	-	-	-	-	-	0.43 (0.26-0.71)	-	Mod
**Surabenjawong 2017**	adm	3mo	4-6	32	1.00 (1.00-1.00)	10	-	-	-	-	-	0.94 (0.91-1.02)	Mod
**Wijdicks 2005**	0-1d	3mo	3-6	60	0.72	-	-	-	-	-	0.84 (0.77-0.92)	0.72	Mod
**Wolf 2007**	0-24hr	30d	3-6	76	-	-	-	-	-	-	0.58 (0.41-0.82)	-	Mod

Abbreviations: FOUR timing, timing of FOUR score assessment relative to the injury date unless stated otherwise; mRS, modified Rankin Scale; Time, timing of mRS assessment unless stated otherwise; Sc, GOS score; Pt, percentage of study population achieving the outcome; AUC, area under receiver operating characteristics curve; Cut-off, cut-off value of FOUR score for logistic regression; Sn, sensitivity; Sp, specificity; PPV, positive predictive value; NPV, negative predictive value; OR, odds ratio in terms of every 1-point increase in FOUR score in relation to achieving the outcome of the study unless stated otherwise; CI, confidence interval; SD, standard deviation;

Timing: adm, on admission; min, minute(s); hr, hour(s); d, day(s); mo, month(s); Dc, discharge;

GCS AUC: Assessment of GCS and outcome at the same time as FOUR score.

Risk of bias: Mod, moderate.

† - Score assessed by consultant

Supplementary Table S5. Results of studies investigating mRS as the outcome.
