# Supplementary material for: The Relationship of the FOUR Score to Patient Outcome: A Systematic Review
Source: J Neurotrauma. 2019 Aug 20;36(17):2469–83. doi: 10.1089/neu.2018.6243 (PMC6709730; doi:10.1089/neu.2018.6243)
Supplement: Supplemental data [file Supp_Table6.pdf]

Neurological (100% neuro causes)				Non-neurological (<50% neuro causes)			
**Study**	**RoB**	**Pt; Causes**	**AUC (95% CI); Outcome / timing**	**AUC (95% CI); Outcome / timing**	**Pt; % of N.Pt**	**RoB**	**Study**
**Chen 2013**	Mod	101; Mixed	0.768 (0.664-0.872); 30d mortality	0.76 (0.67-0.84)[C] 28d mortality	148; 0%	Low	**Rohaut 2017**
**Hu 2017**	Mod	102; Isch stroke	0.819 (0.723-0.883); awareness recovery	0.702 (0.661-0.744); in-hosp mortality	1645; 29.5%	Mod	**Wijdicks 2015**
**Kasprowicz 2016**	Mod	162; TBI	0.906*; in-ICU mortality	0.84* (0.69-0.92); 6mo mortality	85; 5.9%	Mod	**Weiss 2015**
**Mansour 2015**	Mod	127; Isch stroke	0.796 (0.715-0.862); in-hosp mortality	0.837 (0.748–0.926); 28d mortality	86; 15.1%	Mod	**Said 2016**
**Momenyan 2017**	Mod	84; TBI	0.835 (0.739-0.907); in-hosp mortality	0.79 (0.69-0.89); 28d mortality	267; 32.2%	Mod	**Fischer 2010**
**Wijdicks 2005**	Mod	120; Mixed	0.81; in-hosp mortality	-	136; 0%	High	**Fugate 2010**
**Lee 2017**	Mod	105; Stroke	0.758 (0.599-0.916); in-hosp mortality				
**Sadaka 2012**	Mod	51; TBI	0.93; in-hosp mortality				
**Peng 2015**	Mod	120; Mixed	0.834 (0.740-0.928); in-hosp mortality				
**Okasha 2014**	Mod	60; TBI	0.850 (0.734-0.929); in-hosp mortality				
**Zeiler 2017**	Mod	64; SAH	0.762; 1mo mortality				
**Sepahvand 2016**	Mod	198; TBI	0.961; in-hosp mortality				
**Surabenjawong 2017**	Mod	60; Stroke	1.00 (0.94-1.00); 3mo mortality				
**McNett 2016**	Mod	107; TBI	0.813 (0.697-0.928); 6mo GOS1-3				
**Hosseini 2017**	High	80; TBI	0.89 (0.81-0.94); >14d mortality				
**Khanal 2016**	High	97; Mixed	0.82 (0.73-0.91); in-ICU mortality				
**Kocak 2012**	High	100; Stroke	0.675 (0.565-0.786); <15d mortality				
**Gorji 2014**	High	35; TBI	0.92 (0.81-0.97); in-hosp mortality				
**Gorji 2015**	High	80; TBI	0.86 (0.84-0.90); >14d mortality				
**McNett 2014**	High	136; TBI	0.913 (0.822-1.00); in-hosp mortality				
**Babu 2017**	High	98; TBI	0.860; in-hosp mortality				
**Saika 2015**	High	138; TBI	0.97; 14d mortality				
**Stead 2009**	Mod	69; Mixed	-				
**Wolf 2007**	Mod	80; Mixed	-				

Note: Highlighted cells indicate studies of similar timepoints for FOUR and outcome assessments.

Supplementary Table S6. *Comparison between studies comprising of neurological (100% neurological causes) and non-neurological (<50% neurological causes) primary causes of impaired consciousness.*

Neurological (100% neuro causes)				Non-neurological (<50% neuro causes)			
**Study**	**RoB**	**Pt; Causes**	**AUC (95% CI); Outcome / timing**	**AUC (95% CI); Outcome / timing**	**Pt; % of N.Pt**	**RoB**	**Study**
**Zappa 2017**	High	40; Mixed	-				
**Jalali 2014**	High	104; TBI	-				
**Senapathi 2017**	High	63; TBI	-				

Note: Highlighted cells indicate studies of similar timepoints for FOUR and outcome assessments.

Supplementary Table S6 Legend

Abbreviations: RoB, risk of bias; Pt, number of patients; % of N.Pt, percentage of patients with primary neurological causes of impaired consciousness; AUC, area under receiver operating characteristic curve; CI, confidence interval; Outcome / timing, outcome and timepoint of measurement used to calculate the AUC;

RoB: Mod, moderate;

Causes: TBI, traumatic brain injury; Isch, ischaemic; Mixed, mixed neurological causes;

† - these 3 studies are considered to be formed of the same study population.

‡ - the 2016 study is a follow-up of the same cohort reported in 2014.

* - integrated other significant predictors of outcome into the model for calculation

§ - value based on delta day 3-day 1 (i.e. difference in score between day 3 and day 1)

^{C} – c-index value

***Supplementary Table S6** (continued). Comparison between studies comprising of neurological (100% neurological causes) and non-neurological (<50% neurological causes) primary causes of impaired consciousness.*
