# Supplementary material for: The Relationship of the FOUR Score to Patient Outcome: A Systematic Review
Source: J Neurotrauma. 2019 Aug 20;36(17):2469–83. doi: 10.1089/neu.2018.6243 (PMC6709730; doi:10.1089/neu.2018.6243)
Supplement: Supplemental data [file Supp_Table7.pdf]

Neurological ICU				Non-neurological ICU			
**Study**	**RoB**	**Observer**	**AUC (95% CI); Outcome / timing**	**AUC (95% CI); Outcome / timing**	**Observer**	**RoB**	**Study**
**Hu 2017**	Mod	-	0.819 (0.723-0.883); awareness recovery	0.76 (0.67-0.84)[c]; 28d mortality	P	Low	**Rohaut 2017**
**Chen 2013**	Mod	Ne	0.768 (0.664-0.872); 30d mortality	0.906*; in-ICU mortality	-	Mod	**Kasprowicz 2016**
**Peng 2015**	Mod	NsR, N	0.834 (0.740-0.928); in-hosp mortality	0.835 (0.739-0.907) in-hosp mortality	P, N, Stu	Mod	**Momenyan 2017**
**Sadaka 2012**	Mod	Iv	0.93; in-hosp mortality	0.837 (0.748–0.926); 28d mortality	P	Mod	**Said 2016**
**Kocak 2012**	High	Ne	0.675 (0.565-0.786); <15d mortality	0.81; in-hosp mortality	In, R, N	Mod	**Wijdicks 2005**
				0.702 (0.661-0.744); in-hosp mortality	P	Mod	**Wijdicks 2015**
				0.86 in-hosp mortality	N, F, Con#	Mod	**Iyer 2009**
				0.79 (0.69-0.89); 28d mortality	Ne, N, P	Mod	**Fischer 2010**
				0.961; in-hosp mortality	N	Mod	**Sepahvand 2016**
				0.84§ (0.69-0.92); 6mo mortality	In	Mod	**Weiss 2015**
				0.92 (0.81-0.97); in-hosp mortality	Iv	High	**Gorji 2014**
				0.913 (0.822-1.00); in-hosp mortality	Iv	High	**McNett 2014**
				0.89 (0.81-0.94); >14d mortality	-	High	**Hosseini 2017**
				0.82 (0.73-0.91); in-ICU mortality	-	High	**Khanal 2016**
				0.86 (0.84-0.90); >14d mortality	Iv	High	**Gorji 2015**
				-	N, NPsy, ICU Sp, R	Mod	**Bruno 2011**
				-	N	High	**Jalali 2014**
				-	N	Mod	**Wolf 2007**
				-	-	High	**Zappa 2017**

Note : Marcati 2012 and Babu 2017 were excluded due to combination of settings**.** Highlighted cells indicate studies of similar timepoints for FOUR and outcome assessments

Supplementary Table S7*. Comparison between studies comprising of neurological and non-neurological ICU settings.*

Supplementary Table S7 Legend

Abbreviations: RoB, risk of bias; Pt, number of patients; % of N.Pt, percentage of patients with primary neurological causes of impaired consciousness; AUC, area under receiver operating characteristic curve; CI, confidence interval; Outcome / timing, outcome and timepoint of measurement used to calculate the AUC;

RoB: Mod, moderate;

Observer: P, physician(s); N, nurse(s); R, resident(s); NPsy, neuropsychologist(s); Ne, neurologist(s); Iv, investigator(s); F, fellow(s); Con, consultant(s); In, intensivist(s); NsR, neurosurgery resident(s);

† - these 3 studies are considered to be formed of the same study population.

* - integrated other significant predictors of outcome into the model for calculation

§ - value based on delta day 3-day 1 (i.e. difference in score between day 3 and day 1)

# - the observers have never worked in neuroscience ICU or received formal neuroscience training.

[C] – c-index value

Supplementary Table S7 *(continued). Comparison between studies comprising of neurological and non-neurological ICU settings.*
