# Supplementary material for: The Relationship of the FOUR Score to Patient Outcome: A Systematic Review
Source: J Neurotrauma. 2019 Aug 20;36(17):2469–83. doi: 10.1089/neu.2018.6243 (PMC6709730; doi:10.1089/neu.2018.6243)
Supplement: Supplemental data [file Supp_Table8.pdf]

>50% Intubated				0% Intubated			
**Study**	**RoB**	**Pt; % died; % intub**	**AUC (95% CI); Outcome / timing**	**AUC (95% CI); Outcome / timing**	**Pt; % died**	**RoB**	**Study**
**Rohaut 2017**	Low	148; 30%; 100%	0.76 (0.67-0.84)[C]; 28d mortality	0.788(0.722–0.844); in-hosp mortality	185; 14.1%	Low	**Eken 2009**
**Hu 2017**	Mod	102; - 63%	0.819 (0.723-0.883); 90d awareness recovery	0.697[†]; 3mo mortality	100; 24%	Mod	**Gujjar 2013**
**Kasprowicz 2016**	Mod	162; 30.9%; 82.7%	0.906[‡] , SD=0.024; in-ICU mortality	-	104; 36.5%	High	**Jalali 2014**
**Momenyan 2017**	Mod	84; 18.8%; 71.8%	0.835 (0.739-0.907); in-hosp mortality				
**Said 2016**	Mod	86; - 100%	0.837 (0.748–0.926); 28d mortality				
**Chen 2013**	Mod	101; 31.7%; 91%	0.768 (0.664-0.872); 30d mortality				
**Okasha 2014**	Mod	60; 25% 78.3%	0.850 (0.734-0.929); in-hosp mortality				
**Bruno 2011**	Mod	176; - 74%	0.70; 3-mo GOS 1-3				
**Weiss 2015**	Mod	85; - 100%	0.84[§] (0.69-0.92); 6mo mortality;				
**Gorji 2015**	High	80; 18.7%; 100%	0.90 (0.88-0.90); <14d mortality				
**Babu 2017**	High	98; 30.6%; 94%	0.860; in-hosp mortality				
**Zappa 2017**	High	40; - 100%	-				

Note: Highlighted cells indicate studies of similar timepoints for FOUR and outcome assessments.

Supplementary Table S8 Legend

Abbreviations: RoB, risk of bias; Pt, number of patients; % died, percentage of patients who died; % intub, % of patients who were intubated; AUC, area under receiver operating characteristic curve; CI, confidence interval; Outcome / timing, outcome and timepoint of measurement used to calculate the AUC;

RoB: Mod, moderate;

* - integrated other significant predictors of outcome into the model for calculation

§ - value based on delta day 3-day 1 (i.e. difference in score between day 3 and day 1)

[C] – c-index value

Supplementary Table S8. Comparison between studies comprising of mainly intubated patients (>50% intubated) and non-intubated patients (0% intubated).
